# Factors Contributing to Resistance to Ischemia-Reperfusion Injury in Olfactory Mitral Cells

**DOI:** 10.3390/ijms26115079

**Published:** 2025-05-25

**Authors:** Choong-Hyun Lee, Ji Hyeon Ahn, Moo-Ho Won

**Affiliations:** 1 Department of Pharmacy, College of Pharmacy, Dankook University, Cheonan 31116, Republic of Korea; anaphy@dankook.ac.kr; 2 Department of Physical Therapy, College of Health Science, Youngsan University, Yangsan 50510, Republic of Korea; jh-ahn@ysu.ac.kr; 3 Department of Emergency Medicine, Kangwon National University Hospital, Chuncheon 24289, Republic of Korea; 4 Department of Emergency Medicine, School of Medicine, Kangwon National University, Chuncheon 24341, Republic of Korea

**Keywords:** neuronal survival, excitotoxicity, antioxidant enzymes, metabolic failure, neuroprotective signaling pathways, microvascular architecture

## Abstract

Brain ischemia-reperfusion (IR) injury is a critical pathological process that leads to extensive neuronal death, with hippocampal pyramidal cells, particularly those in the cornu Ammonis 1 (CA1) subfield, being highly vulnerable. Until now, human olfactory mitral cell resistance to IR injury has not been directly studied, but olfactory dysfunction in humans is frequently reported in systemic vascular conditions such as ischemic heart failure and may serve as an early clinical marker of neurological or cardiovascular disease. Mitral cells, the principal neurons of the olfactory bulb (OB), exhibit remarkable resistance to IR injury, suggesting the presence of unique molecular adaptations that support their survival under ischemic stress. Several factors may contribute to the resilience of mitral cells. They have a lower susceptibility to excitotoxicity, mitigating the harmful effects of excessive glutamate signaling. Additionally, they maintain efficient calcium homeostasis, preventing calcium overload—a major trigger for cell death in vulnerable neurons. Mitral cells may also express high baseline levels of antioxidant enzymes and their activities, counteracting oxidative stress. Their robust mitochondrial function enhances energy production and reduces susceptibility to metabolic failure. Furthermore, neuroprotective signaling pathways, including phosphatidylinositol-3-kinase (PI3K)/Akt, mitogen-activated protein kinase/extracellular signal-regulated kinase (MAPK/ERK), and nuclear factor erythroid-2-related factor 2 (Nrf2)-mediated antioxidative responses, further bolster their resistance. In addition to these intrinsic mechanisms, the unique microvascular architecture and metabolic support within the olfactory bulb provide an extra layer of protection. By comparing mitral cells to ischemia-sensitive neurons, key vulnerabilities—such as oxidative stress, excitotoxicity, calcium dysregulation, and mitochondrial dysfunction—can be identified and potentially mitigated in other brain regions. Understanding these molecular determinants of neuronal survival may offer valuable insights for developing novel neuroprotective strategies to combat IR injury in highly vulnerable areas, such as the hippocampus and cortex.

## 1. Introduction

The olfactory system is responsible for detecting and processing odor information and plays a vital role in sensory perception [1,2]. The system consists of peripheral components (the olfactory epithelium and olfactory nerve), central components (the olfactory bulb and olfactory tract), and higher-order processing areas (the olfactory cortex, amygdala, entorhinal cortex, hippocampus, and orbitofrontal cortex [1]. It begins with olfactory receptor neurons in the nasal epithelium, which transmit signals to the olfactory bulb (OB) [1]. Within the OB, mitral cells, as the principal output neurons, integrate sensory signals and refine odor information through interactions with tufted cells, granule cells, and periglomerular cells, which provide inhibitory modulation [2,3].

Brain or cerebral ischemia, which is characterized by reduced blood flow to neural tissue, leads to oxygen and nutrient deprivation and causes cellular dysfunction and death [4,5]. Reperfusion injury occurs when blood flow is restored, which contributes to secondary brain damage after ischemic stroke or cardiac arrest, often exacerbating damage through excitotoxicity, oxidative stress, and inflammation [4,5]. Most central neurons are highly vulnerable to these conditions, resulting in severe neurological impairments [6].

**Figure 1:**
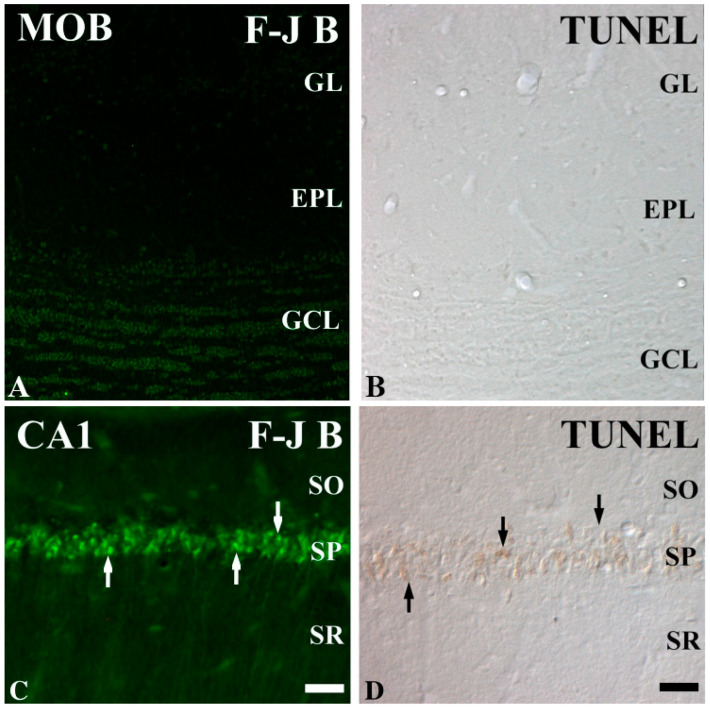
Fluoro Jade B (F-J B) histofluorescence and terminal deoxynucleotidyl transferase dUTP nick end labeling (TUNEL) staining in the main olfactory bulb (MOB; (**A**,**B**)) and hippocampal CA1 region (**C**,**D**) of the gerbil. F-J B and TUNEL-positive cells are rare in the MOB of the IR group, but many F-J B (white arrows) and TUNEL (black arrows)-positive cells are shown in the CA1 region after IR injury. EPL external plexiform layer; GCL granule cell layer; GL glomerular layer; SO stratum radiatum, SP stratum pyramidale; SR stratum radiatum. Scale Bars = 200 μm (**A**,**B**), 50 μm (**C**,**D**). This figure was published by Choi et al. (2010) [12].

IR injury typically results in extensive neuronal damage in brain regions such as the hippocampus, but emerging studies in animal models (e.g., gerbils, rats, dogs) suggest that mitral cells in the olfactory bulb may exhibit resistance [7,8,9,10,11,12] (Figure 1). Investigating the mechanisms underlying their resilience could provide critical insights into neuroprotection, potentially guiding therapeutic approaches for ischemic stroke and other ischemic brain disorders. In addition, while mitral cell resistance to IR injury has been demonstrated in rodent and canine models [7,8,10,13,14], extrapolation to the human brain must be performed cautiously due to known species-specific differences in olfactory bulb structure, vascularization, and neural connectivity [1,13]. Although mitral cell morphology differs across species, the evolutionary conservation of redox homeostatic mechanisms [14] and the identification of a rodent-like neurogenic migratory system in primates [15] support the translational relevance of rodent models. This review aims to synthesize current evidence from these animal studies and explore molecular and anatomical factors contributing to this resilience, while acknowledging that extrapolation to human biology must be performed cautiously due to species-specific differences in olfactory system anatomy and function.

For the literature search strategy, we conducted a focused literature search using PubMed, Scopus, and Google Scholar databases. The keywords included: olfactory mitral cells, ischemia-reperfusion injury, neuronal resistance, oxidative stress, mitochondrial function, antioxidant enzymes, and neuroprotection. Emphasis was placed on original experimental research, reviews, and histological reports that compared IR-resistant and IR-vulnerable neurons.

## 2. Structure and Function of Olfactory Mitral Cells

**Figure 2:**
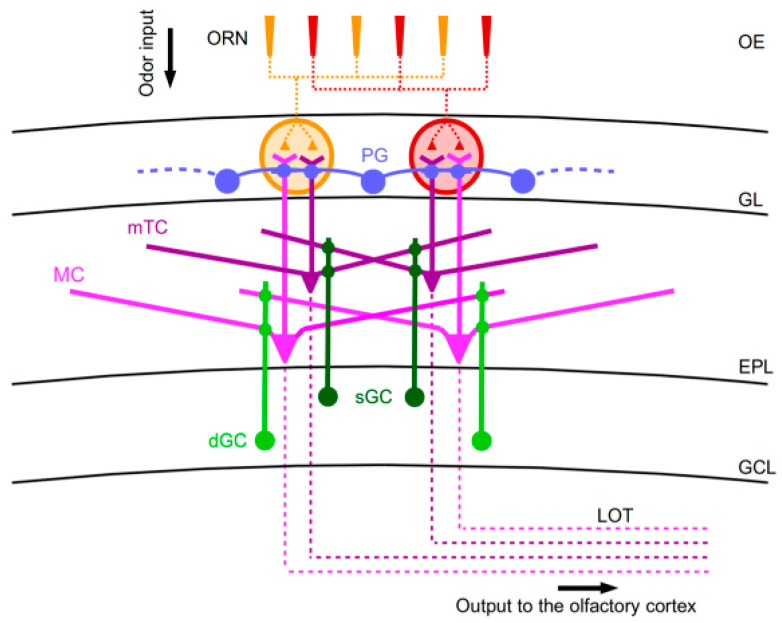
Schematic representation of the translaminar organization of the olfactory bulb (OB) and its position. The olfactory receptor (or sensory) neurons (ORN) in the olfactory epithelium (OE) project to the glomerular cell layer (GL), where they connect to periglomerular (PG), as well as mitral (MC) and middle tufted (mTC) cells, which connect to granule cells (GC). The OB output propagates to the olfactory cortex through the MC and mTC axons in the lateral olfactory tract (LOT). EPL, external plexiform layer; GCL, granule cell layer. This figure was published by Cavarretta et al. (2018) [16].

In the OB, mitral cells are a principal type of excitatory neuron and play a crucial role in processing olfactory information. They receive direct input from olfactory receptor neurons, which are located in the nasal cavity, via synapses in the glomeruli of the OB, where each mitral cell connects to a specific set of sensory inputs [1,3] (Figure 2). Anatomically, mitral cells have large, apical dendrites that extend into the glomerular layer and basal dendrites that interact with local interneurons, such as granule cells, in the external plexiform layer [2] (Figure 2). Their axons project to higher-order brain regions (the piriform cortex, amygdala, entorhinal cortex, etc.), forming the olfactory pathway that links sensory perception to cognitive and emotional responses [2] (Figure 2).

Mitral cells are essential for olfactory signal processing as the primary relay between sensory input and higher cortical areas [1,2,3] as follows. The mitral cells receive excitatory input from olfactory sensory neurons and refine the signal through interactions with inhibitory interneurons, particularly periglomerular and granule cells. They exhibit synchronized oscillatory activity, which is crucial for encoding odor identity and intensity. Finally, they ensure, through their widespread projections, that olfactory information is integrated with memory and emotional processing centers, allowing for the perception and interpretation of complex odor stimuli. In addition, ultrastructural studies of olfactory mitral cells have revealed several unique morphological features that may support their functional resilience. Electron microscopy shows that mitral cells contain abundant, elongated mitochondria clustered near dendritic and axonal synapses, suggesting a high capacity for localized energy production and calcium buffering [17,18]. These mitochondria are often tightly associated with smooth and rough endoplasmic reticulum, forming mitochondrial-associated membranes (MAMs) that facilitate lipid transfer and calcium signaling—both of which are critical for stress responses [19,20]. The nuclei of mitral cells are typically large and euchromatic, reflecting high transcriptional activity, while their somatic cytoplasm is rich in ribosomes and Golgi apparatus, indicating robust protein synthesis [21]. In contrast to ischemia-sensitive neurons such as hippocampal CA1 pyramidal cells—which show early mitochondrial swelling and fragmentation following IR injury—mitral cell mitochondria appear more structurally stable, even under ischemic stress [14]. These ultrastructural attributes may underlie their superior bioenergetic function and lower susceptibility to ischemic damage.

When compared to other central nervous system neurons, mitral cells possess several unique properties that contribute to their specialized function. One of their remarkable characteristics is their resistance to ischemia [8,9,10,11], a feature that distinguishes them from vulnerable neurons in regions such as the hippocampus [22] and cortex [23]. In addition, the OB may possess evolutionary adaptations that confer enhanced resistance to IR injury, particularly in rodents. In many rodent species, the OB plays a central role in environmental navigation, food foraging, predator detection, and social communication, making olfaction their dominant sensory modality [24,25]. This reliance on olfaction likely exerted evolutionary pressure to preserve OB function under a variety of stress conditions, including transient metabolic deprivation. Supporting this idea, olfactory structures in rodents exhibit high vascular density, robust metabolic support, and relatively consistent perfusion even under systemic stress [26,27]. Moreover, rodents typically show a lower incidence of cerebrovascular events compared to humans, possibly reflecting species-specific cerebrovascular patterns and adaptive resistance mechanisms in critical brain regions like the OB [28,29]. These considerations suggest that the IR resistance observed in mitral cells may be the result of evolutionary optimization to protect a functionally dominant brain structure in species highly dependent on olfaction. Taken together, the resilience may be attributed to differences in metabolic demand, ion channel composition, or neuroprotective mechanisms, which are discussed in this review.

## 3. Mechanisms of IR Injury in the Brain

**Figure 3:**
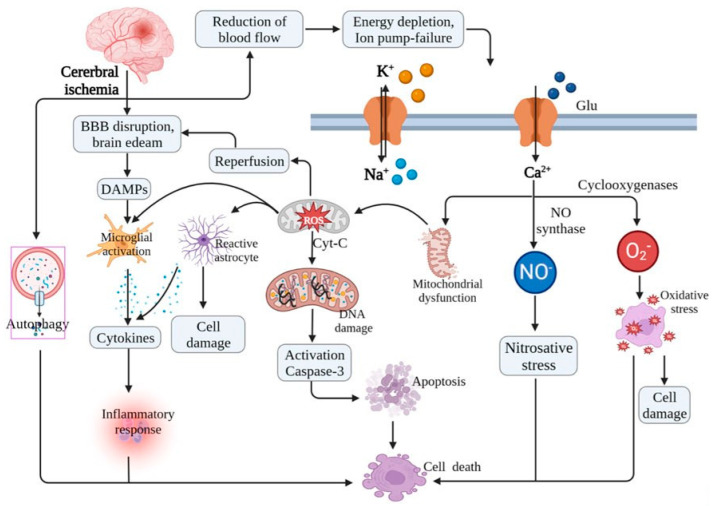
Mechanisms of brain IR injury. This figure represents the cascade of pathological events occurring during IR injury. The interplay between excitotoxicity, oxidative stress, inflammation, and mitochondrial dysfunction contributes to neuronal death and brain damage. Excessive Ca^2+^ influx and ROS lead to mitochondrial dysfunction and the opening of the mitochondrial permeability transition pore (mPTP). Cytochrome C is released into the cytoplasm and activates caspases, which drive apoptosis. This figure was originally published by Zheng et al. (2023) [6].

IR injury in the brain is a complex pathological process involving a cascade of biochemical and molecular events. Ischemic stroke occurs when cerebral blood flow is reduced due to an obstruction in the arteries and leads to oxygen and glucose deprivation [30]. When blood flow is restored, reperfusion can paradoxically exacerbate neuronal damage through excitotoxicity, oxidative stress, inflammation, and mitochondrial dysfunction [31] (Figure 3). Excitotoxicity occurs during ischemia as follows [32,33]. ATP production is reduced due to mitochondrial dysfunction leading to intracellular acidosis, ion pump failure, and the accumulation of intracellular sodium and calcium. To compensate for acidosis, the Na^+^/H^+^ exchanger is activated, resulting in Na^+^ influx and increased intracellular Na^+^ levels [34]. Simultaneously, impaired Na^+^/K^+^-ATPase function further exacerbates intracellular Na^+^ accumulation. These changes trigger the reverse operation of the Na^+^/Ca^2+^ exchanger, leading to Ca^2+^ overload. This loss of ionic homeostasis results in excessive glutamate release and sustained membrane depolarization, thereby initiating excitotoxicity. The overactivation of N-methyl-D-aspartate (NMDA) and alpha-amino-3-hydroxy-5-methyl-4-isooxazole-propionic acid (AMPA) receptors facilitates further calcium influx, which activates calpain, phospholipases, and other proteases that degrade cellular components and contribute to neuronal death.

Oxidative stress occurs following reperfusion as follows. Reperfusion introduces a sudden influx of oxygen, which promotes the generation of reactive oxygen species (ROS), including superoxide anions and hydroxyl radicals. Excessive ROS production damages lipids, proteins, and DNA, leading to apoptosis and necrosis [30,35].

Inflammation and blood-brain barrier (BBB) disruption after/during reperfusion occur as follows [36,37]. Reperfusion activates microglia and astrocytes, which leads to the release of pro-inflammatory cytokines such as tumor necrosis factor-alpha (TNF-α) and interleukins (IL-1β, IL-6). In addition, infiltrating neutrophils exacerbate inflammation by producing matrix metalloproteinases (MMPs), which degrade tight junction proteins, leading to BBB disruption, vasogenic edema, and hemorrhagic transformation.

During reperfusion, mitochondrial permeability transition pore (mPTP) opening leads to the release of cytochrome c and caspase activation, which promotes apoptosis. Additionally, excessive calcium and ROS contribute to mitochondrial collapse and neuronal death [31,38].

## 4. Types of Neurons Vulnerable and Resistant to IR Injury

Neurons exhibit varying degrees of vulnerability to IR injury. As described above, the vulnerability is influenced by factors such as metabolic demand, cellular structure, and blood supply. Neurons in the hippocampus, such as CA1 pyramidal cells, are highly susceptible due to their complex dendritic arborizations and dependence on high metabolic activity [39]. Purkinje cells, as principal neurons in the cerebellum, are highly vulnerable to IR injury, likely due to their high metabolic demand [40]. Conversely, certain neuron types display resistance to IR injury, notably olfactory mitral cells, which are protected by unique cellular features, such as lower excitability, specialized vascularization, and metabolic support. Overall, the response of neurons to IR injury is determined by a combination of anatomical, physiological, and molecular factors, with some cells exhibiting remarkable resistance to damage. As shown in Table 1, according to the types of neurons, they are known to be vulnerable or resistant to IR injury.

## 5. Factors of Mitral Cell Resistance to IR Injury

Olfactory mitral cells exhibit remarkable resistance to IR injury compared to other central nervous system (CNS) neurons, as described above. The following unique biochemical adaptation may contribute to mitral cells’ survival under ischemic stress, offering insights into potential neuroprotective strategies for vulnerable neuronal populations, such as hippocampal pyramidal cells, in ischemic brain injury.

### 5.1. Low Excitotoxicity Susceptibility and Efficient Calcium Homeostasis

In brain IR injury, excitotoxicity, as a major mechanism of neuronal death/loss, is primarily driven by excessive glutamate release and overstimulation of ionotropic glutamate receptors [46]. However, not all neurons exhibit the same degree of susceptibility to excitotoxic damage [47,48]. Upon reviewing the available literature, studies directly linking the low excitotoxicity susceptibility of olfactory mitral cells to their resistance against IR injury are limited or not readily accessible [49,50]. Nevertheless, one potential factor contributing to the resilience of mitral cells to IR injury is their relatively low NMDA receptor (NMDAR) density compared to highly vulnerable neuronal populations, such as hippocampal CA1 pyramidal neurons [51]. In addition, mitral cells receive extensive inhibitory input from granule and periglomerular cells, which regulate excitatory signaling and may help prevent sustained glutamate receptor activation and calcium overload [52,53,54]. Another important factor is the intrinsic calcium homeostasis of mitral cells. When compared to highly vulnerable neurons, mitral cells may have more efficient calcium buffering mechanisms, including enhanced mitochondrial calcium uptake or increased expression of calcium-binding proteins, both of which can limit excitotoxic damage [55,56]. Taken together, these findings suggest that mitral cells possess multiple intrinsic and network-level features that reduce their susceptibility to excitotoxic injury. However, direct experimental evidence confirming these mechanisms remains limited. Furthermore, although NMDA receptors are well-known mediators of excitotoxicity during IR injury due to their high calcium permeability, they are also essential for physiological processes such as learning and memory through their role in synaptic plasticity and long-term potentiation [57]. Therefore, broad inhibition of NMDA receptors—while neuroprotective in the short term—can impair cognitive function if used indiscriminately [58]. This dual role underscores the complexity of targeting NMDA signaling therapeutically. In olfactory mitral cells, lower baseline NMDA receptor density may offer a protective advantage during IR injury without entirely abolishing their synaptic plasticity or integrative function. Therefore, neuroprotective approaches may benefit from selectively targeting receptor subtypes or modulating temporal activation windows to reduce excitotoxicity while preserving memory-related functions [59]. Taken together, future studies should focus on electrophysiological and molecular analyses of glutamate receptor activity, inhibitory synaptic regulation, and calcium homeostasis in mitral cells following IR injury. Understanding these properties may provide valuable insights into neuronal resilience and potential neuroprotective strategies.

### 5.2. High Antioxidant Levels or Activities

Both the levels and activities of antioxidant enzymes play critical roles in neuronal resistance to IR injury, but enzyme activity is generally more critical for neuroprotection [60,61]. Upon reviewing the available literature, it appears that specific studies directly linking the high antioxidant enzyme levels of olfactory mitral cells to their resistance against IR injury are limited or not readily accessible [62]. While general mechanisms of neuronal resistance to oxidative stress have been documented, direct evidence pertaining to olfactory mitral cells is scarce. One potential factor contributing to this resilience is the high baseline expression of antioxidant enzymes, such as superoxide dismutase (SOD), catalase, and glutathione peroxidase, which neutralize reactive oxygen species (ROS) and mitigate oxidative stress [63]. Oxidative stress plays a critical role in IR-induced neuronal death, particularly in hippocampal CA1 pyramidal neurons, which have lower endogenous antioxidant defenses and are highly susceptible to excitotoxicity and mitochondrial dysfunction [61,64,65]. Taken together, it is suggested that antioxidant-rich environments protect neurons from ischemic damage, and mitral cells may inherently maintain a more robust antioxidant system, reduce ROS accumulation, and preserve cellular integrity. In the future, we need studies on changes in levels of antioxidant enzymes or their activities in the olfactory mitral cells following IR injury.

### 5.3. Robust Mitochondrial Function

Hippocampal CA1 pyramidal neurons, which are highly vulnerable to IR injury, display significant mitochondrial dysfunction, including impaired calcium handling and increased susceptibility to oxidative damage [66,67]. Mitochondria play a crucial role in neuronal survival and function, particularly during IR injury, where energy failure and oxidative stress contribute to neuronal damage. One of the key determinants of neuronal resistance to IR injury is mitochondrial integrity and efficiency in energy production, calcium buffering, and ROS regulation [68,69]. While direct studies on mitochondrial function in olfactory mitral cells following IR injury remain limited, evidence suggests that mitral cells may possess a robust mitochondrial network that enhances their resilience. One potential factor contributing to this resistance is the high density of mitochondria within mitral cells, which can support sustained synaptic activity and ATP production under metabolic stress [70,71]. Additionally, mitral cells may exhibit a more efficient oxidative phosphorylation system, reducing the likelihood of ATP depletion and mitochondrial depolarization, which are critical events in IR-induced neuronal death [72,73]. Finally, Mitochondrial uncoupling proteins (UCPs), such as UCP2, UCP4, and UCP5, are known to play neuroprotective roles by mitigating ROS generation and preserving mitochondrial membrane potential [74,75,76,77]. Although specific studies in mitral cells are lacking, the possible involvement of UCPs may contribute to the robust mitochondrial function observed in these cells under ischemic conditions. Taken together, these findings suggest that robust mitochondrial function, efficient ATP production, and enhanced mitochondrial resilience may underlie the lower susceptibility of mitral cells to IR-induced damage. Future studies should investigate mitochondrial dynamics, bioenergetic profiles, and oxidative stress responses in olfactory mitral cells following ischemic events to better understand their unique neuroprotective mechanisms.

### 5.4. Neuroprotective Signaling Pathways

Neuronal survival following IR injury is highly dependent on the activation of protective intracellular signaling pathways that regulate oxidative stress, inflammation, and apoptosis [78,79]. Studies have identified key signaling cascades, including the PI3K/Akt pathway, MAPK/ERK signaling, and Nrf2-mediated antioxidative responses, as critical determinants of neuronal resistance to ischemic damage [79,80,81]. However, direct evidence regarding the activation of these pathways in olfactory mitral cells remains limited. The PI3K/Akt pathway is a well-documented prosurvival mechanism that promotes neuronal resilience by inhibiting apoptosis and enhancing metabolic stability under stress conditions [79]. Given the relative resistance of mitral cells to IR-induced apoptosis, it is plausible that these neurons exhibit sustained Akt activation during ischemic stress. Another pathway is MAPK/ERK signaling, which plays a dual role in neuronal survival and plasticity. ERK1/2 activation is associated with neuroprotection by modulating gene expression, increasing antioxidant defenses, and reducing excitotoxic damage [80,82]. Since mitral cells demonstrate lower susceptibility to excitotoxicity compared to hippocampal CA1 pyramidal neurons, persistent ERK activity may underlie their enhanced ischemic tolerance. In addition, the Nrf2/ARE pathway is a crucial regulator of antioxidant gene expression and is essential for mitigating oxidative stress during IR injury [81]. Activation of Nrf2 enhances the transcription of antioxidant enzymes such as heme oxygenase-1 (HO-1), NAD(P)H quinone oxidoreductase-1 (NQO1), and glutathione S-transferase, which collectively reduce ROS accumulation and cellular damage [83,84]. While Nrf2 signaling is well-characterized in other neuronal populations, its specific role in mitral cell resilience warrants further investigation. Based on those findings, future studies should focus on characterizing the activation profiles of PI3K/Akt, MAPK/ERK, and Nrf2 pathways in mitral cells following IR injury, as these could provide insight into novel therapeutic targets for neuroprotection.

### 5.5. Unique Microvascular and Metabolic Support

**Figure 4:**
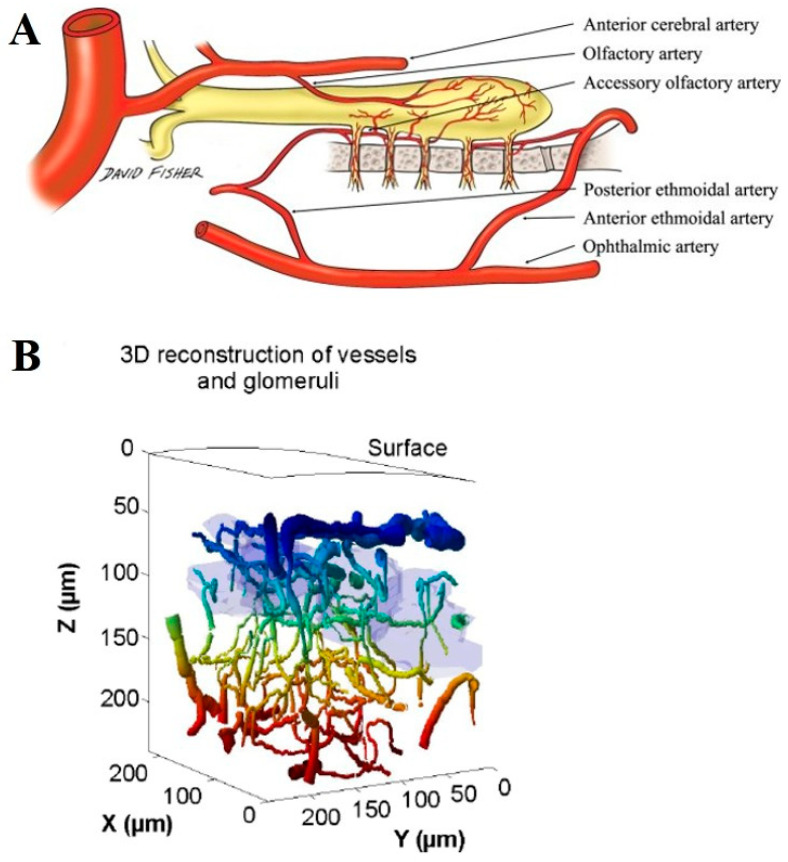
(**A**) Arterial supply of the OB and olfactory nerve. The OB is mainly supplied by the olfactory artery, a branch of the anterior cerebral artery. This figure was published by Hendrix et al. (2014). (**B**) Three-dimensional reconstruction of vessels and glomeruli. Vessel colors vary with depth, and glomeruli are indicated in light blue. This figure was published by Lecoq et al. (2009) [26].

OB exhibits a distinct vascular architecture and metabolic profile that may contribute to the resilience of mitral cells against IR injury. Compared to other brain regions, the OB has a relatively high capillary density and unique patterns of blood flow regulation, which may enhance oxygen and nutrient delivery even under conditions of transient ischemia [26,27,85,86] (Figure 4). This well-developed microvascular network could facilitate more efficient metabolic support and reduce the severity of ischemic damage. One key factor in the metabolic resilience of mitral cells is their ability to efficiently utilize aerobic metabolism and maintain mitochondrial integrity. Studies have shown that neurons with robust mitochondrial function are better equipped to handle oxidative stress and energy depletion following IR injury [87,88]. The OB also possesses a high metabolic rate, which may be supported by enhanced glucose uptake and lactate metabolism from surrounding glial cells, particularly astrocytes [89,90]. Astrocyte-derived lactate can serve as an alternative energy substrate for neurons under metabolic stress, thereby preserving mitral cell function during IR episodes. Furthermore, the OB is uniquely equipped with high levels of neurovascular coupling and ensures rapid adjustments in blood flow in response to metabolic demands [91,92]. This mechanism may provide mitral cells with a more stable energy supply and reduce the impact of ischemic insults. Additionally, the presence of specialized pericytes and endothelial cells in olfactory bulb capillaries, which can modulate cerebral blood flow by changing capillary diameter, may enhance BBB integrity and facilitate neuroprotective signaling [91,93]. Overall, the unique microvascular and metabolic adaptations of OB likely play a crucial role in protecting mitral cells from IR-induced damage. Future studies should investigate the specific contributions of these adaptations to mitral cell survival and determine whether they can be leveraged for therapeutic strategies against cerebral ischemic insults.

## 6. Implications for Neuroprotection and Therapeutic Potential

The unique resilience of olfactory mitral cells to IR injury offers valuable insights into potential neuroprotective strategies for vulnerable brain regions. When we understand the molecular adaptations that confer resistance to mitral cells, we can explore targeted interventions to enhance neuronal survival following ischemic events. Several key mechanisms may serve as therapeutic targets as follows. The reduced susceptibility of mitral cells to glutamate-induced excitotoxicity suggests that therapies aimed at modulating glutamate receptors or enhancing glutamate clearance may help protect vulnerable neurons, such as hippocampal CA1 pyramidal cells, from IR injury. Maintaining calcium balance is crucial for neuronal survival. Strategies that enhance calcium buffering capacity, such as upregulating calcium-binding proteins or modulating ion channels, could mitigate calcium overload and associated cytotoxic effects in ischemia-sensitive neurons, such as hippocampal CA1 pyramidal neurons. The high baseline expression of antioxidant enzymes in mitral cells underscores the potential benefits of antioxidant-based therapies. Upregulating endogenous antioxidant responses through Nrf2 activators or administering exogenous antioxidants may reduce oxidative damage and improve neuronal resilience. Given the robust mitochondrial function of mitral cells, interventions that enhance mitochondrial bioenergetics—such as mitochondrial-targeted antioxidants, metabolic substrates, or pharmacological agents that stabilize mitochondrial integrity—could support neuronal survival in ischemic conditions. Targeting key protective signaling cascades, including PI3K/Akt and MAPK/ERK, could provide broad-spectrum neuroprotection. Pharmacological agents that activate these pathways may help sustain cell survival and function in ischemia-prone regions. The OB’s specialized vascular and metabolic environment highlights the importance of optimizing cerebral blood flow and energy supply. Strategies such as promoting angiogenesis, enhancing cerebral perfusion, and modulating metabolic substrates may improve neuronal survival after IR injury.

## 7. Future Directions and Open Questions

Several critical questions remain unanswered despite the understanding of mitral cell resilience to IR injury. Addressing these knowledge gaps may further enhance our ability to develop effective neuroprotective strategies: Identifying key genes, transcriptional regulators, and epigenetic modifications that contribute to their resilience could provide new therapeutic targets. Investigating the role of specialized vascular structures, BBB integrity, and metabolic coupling in sustaining mitral cells could inform strategies to enhance perfusion and nutrient supply in ischemia-sensitive regions. Determining whether ischemia-prone neurons can be reprogrammed or conditioned to adopt similar resistance mechanisms could open new avenues for neuroprotection. Understanding whether mitral cells exhibit unique interactions with glial cells or inflammatory mediators may reveal novel anti-inflammatory strategies for mitigating IR injury. Investigating whether mitral cells sustain subtle impairments post-ischemia and how they recover functionally over time may provide insights into long-term neuronal resilience. Furthermore, despite evidence from animal models indicating that mitral cells are structurally resistant to IR injury, olfactory dysfunction is widely recognized as an early biomarker of neurodegenerative diseases, including Parkinson’s and Alzheimer’s disease [13,94]. In addition, although human olfactory mitral cell resistance has not been directly studied, olfactory dysfunction in humans is frequently reported in systemic vascular conditions such as ischemic heart failure and may serve as an early clinical marker of neurological or cardiovascular disease [95,96]. This apparent discrepancy may reflect the fact that olfactory deficits can arise from functional alterations—such as synaptic changes, glial activation, and axonal degeneration—rather than overt neuronal loss [97], which deserves future investigation. Thus, mitral cells may survive IR insults structurally while experiencing sublethal dysfunction that contributes to clinical olfactory decline. These findings suggest that resilience to IR injury does not exclude the possibility of early olfactory impairment in neurodegenerative conditions and highlight the need to distinguish between neuronal viability and function in disease models. Taken together, future research should focus on mechanistic studies, in vivo models, and translational approaches to apply these findings to clinical settings. Addressing these open questions will be essential for advancing neuroprotective strategies against ischemic brain injury.

## 8. Conclusions

Olfactory mitral cells exhibit strong resistance to IR injury, which distinguishes them from other neuronal populations that are highly vulnerable to ischemic damage. Their resilience might be attributed to multiple intrinsic and extrinsic factors, including reduced excitotoxicity susceptibility, efficient calcium homeostasis, robust antioxidant defenses, strong mitochondrial function, and activation of neuroprotective signaling pathways. Additionally, the specialized vascular and metabolic environment of the OB provides further support for mitral cell survival. Understanding these protective mechanisms not only deepens our knowledge of neuronal ischemia resistance, but also holds significant therapeutic potential for treating IR injury in vulnerable brain regions. By leveraging insights from mitral cells, novel neuroprotective strategies—ranging from pharmacological interventions to gene therapy—could be developed to enhance neuronal survival following ischemic events. However, several critical questions remain, necessitating further research to fully elucidate the molecular and physiological basis of mitral cell resilience. By translating these findings into clinical applications, the field of neuroprotection may advance toward more effective therapies for ischemic stroke and other conditions characterized by neuronal vulnerability to ischemic stress.

## Figures and Tables

**Table 1:** Types of neurons vulnerable and resistant to IR injury.

Neuron Type	Vulnerability to IR Injury	Location	Primary Function	References
CA1 pyramidal neurons	Highly vulnerable	Hippocampus (CA1 region)	Long-term memory formation, synaptic plasticity	[39]
Cerebellar Purkinje Cells	Highly vulnerable	Cerebellum	Coordination of movement, motor learning	[40]
Neocortical Pyramidal Neurons	Moderately Vulnerable	Neocortex (Layer 5)	Motor control, cognition, sensory processing	[41]
Dopaminergic Neurons	Resistant	Substantia Nigra, Ventral Tegmental Area	Motor control, reward processing, mood regulation	[42]
Spinal Motor Neurons	Resistant	Spinal Cord	Motor function, voluntary movement	[40,43]
Striatal Interneurons	Resistant	Striatum	Regulation of motor activity, reward and emotional processing	[44]
Dentate granule cells	Highly resistant	Dentate gyrus	Learning, memory, and spatial navigation	[45]
Olfactory mitral cells	Resistant	Olfactory Bulb	Olfaction, processing odor signals	[9]

## Data Availability

Data sharing not applicable.

## Abbreviations

alpha-amino-3-hydroxy-5-methyl-4-isooxazole-propionic acid receptor (AMPAR), blood-brain barrier (BBB), central nervous system (CNS), cornu Ammonis (CA), interleukin (IL), Fluoro Jade B (F-J B), heme oxygenase-1 (HO-1), ischemia-reperfusion (IR), mitochondrial-associated membranes (MAMs), matrix metalloproteinases (MMPs), mitochondrial permeability transition pore (mPTP), mitogen-activated protein kinase/extracellular signal-regulated kinase (MAPK/ERK), NAD(P)H quinone oxidoreductase-1 (NQO1), N-methyl-D-aspartate receptor (NMDAR), nuclear factor erythroid-2-related factor 2 (Nrf2); olfactory bulb (OB), phosphatidylinositol-3-kinase (PI3K), reactive oxygen species (ROS); superoxide dismutase (SOD), terminal deoxynucleotidyl transferase dUTP nick end labeling (TUNEL), tumor necrosis factor-alpha (TNF-α).
